# Neighborhood Disadvantage and Breast Cancer–Specific Survival in the US

**DOI:** 10.1001/jamanetworkopen.2024.7336

**Published:** 2024-04-18

**Authors:** Neha Goel, Alexandra E. Hernandez, Angela Mazul

**Affiliations:** 1 Department of Surgery, Division of Surgical Oncology, University of Miami Miller School of Medicine, Miami, Florida; 2 Sylvester Comprehensive Cancer Center, University of Miami Miller School of Medicine, Miami, Florida; 3 Harvard T.H. Chan School of Public Health, Harvard University, Boston, Massachusetts; 4 University of Pittsburgh Medical Center Hillman Cancer Center, Pittsburgh, Pennsylvania; 5 Department of Otolaryngology, University of Pittsburgh Medical Center, Pittsburgh, Pennsylvania; 6 Washington University School of Medicine, St Louis, Missouri

## Abstract

**Importance:**

Despite improvements in breast cancer screening, treatment, and survival, disparate breast cancer–specific survival outcomes persist, particularly in disadvantaged neighborhoods. Most of these disparities are attributed to disparities in individual, tumor, and treatment characteristics. However, a critical knowledge gap exists as to whether disparities in breast cancer–specific survival remain after accounting for individual, tumor, and treatment characteristics.

**Objective:**

To evaluate if neighborhood disadvantage is associated with shorter breast cancer–specific survival after controlling for individual, tumor, and treatment characteristics in a national population.

**Design, Setting, and Participants:**

This national retrospective cohort study included patients with breast cancer diagnosed from 2013 to 2018 from the Surveillance, Epidemiology, and End Results 17 Census tract–level socioeconomic status and rurality database of the National Cancer Institute. Data analysis was performed from September 2022 to December 2023.

**Exposures:**

Neighborhood disadvantage measured by Yost index quintiles.

**Main Outcomes and Measures:**

Breast cancer–specific survival was evaluated using a competing risks cause-specific hazard model controlling for age, race, ethnicity, rurality, stage, subtype, insurance, and receipt of treatment.

**Results:**

A total of 350 824 patients with breast cancer were included; 41 519 (11.8%) were Hispanic, 39 631 (11.3%) were non-Hispanic Black, and 234 698 (66.9%) were non-Hispanic White. A total of 87 635 patients (25.0%) lived in the most advantaged neighborhoods (group 5) and 52 439 (14.9%) lived in the most disadvantaged neighborhoods (group 1). A larger number of non-Hispanic White patients (66 529 patients [76.2%]) lived in advantaged neighborhoods, while disadvantaged neighborhoods had the highest proportion of non-Hispanic Black (16 141 patients [30.9%]) and Hispanic patients (10 168 patients [19.5%]). Breast cancer–specific survival analysis found the most disadvantaged neighborhoods (group 1) had the highest risk of mortality (hazard ratio, 1.43; 95% CI, 1.36-1.50; *P* < .001) compared with the most advantaged neighborhoods.

**Conclusions and Relevance:**

In this national cohort study of patients with breast cancer, neighborhood disadvantage was independently associated with shorter breast cancer–specific survival even after controlling for individual-level factors, tumor characteristics, and treatment. This suggests potential unaccounted-for mechanisms, including both nonbiologic factors and biologic factors.

## Key Points

**Question:**

Do neighborhood-level disparities in breast cancer–specific survival remain after accounting for individual, tumor, and treatment characteristics?

**Findings:**

This cohort study of 350 824 patients identified that neighborhood disadvantage was independently associated with shorter breast cancer–specific survival even after controlling for individual, tumor, and treatment characteristics.

**Meaning:**

These findings suggest that persistent disparities in survival reflect potential unaccounted-for social and biological mechanisms through which neighborhood disadvantage leads to shorter breast cancer–specific survival.

## Teaser

This cohort study evaluates if neighborhood disadvantage is associated with shorter breast cancer–specific survival after controlling for individual, tumor, and treatment characteristics in a national population.

## Introduction

Despite improvements in breast cancer screening, treatment, and survival, disparate outcomes persist, particularly in disadvantaged neighborhoods. Neighborhoods reflect complex environments with unique cultural, physical, and economic attributes, and disadvantaged neighborhoods contribute to the creation and persistence of under-resourced neighborhoods limiting access to care such as screening mammography and treatment, ultimately impacting breast cancer survival.^1,2,3,4,5,6^ While many of these survival disparities are secondary to access to care barriers, recent literature has shown breast cancer–specific survival disparities exist after controlling for these measures. Specifically, studies have shown associations with neighborhood disadvantage, lower neighborhood socioeconomic status, and geospatial measures of economic residential segregation with shorter breast cancer survival after controlling for many of the individual-level, neighborhood-level, and structural factors that have been associated with shorter breast cancer survival in previous literature. This residual association of neighborhood disadvantage with survival suggests that there are underlying mechanisms by which neighborhood disadvantage impacts survival that have not been accounted for.^7,8^ Many of these studies are limited to specific geographic areas, limiting the generalizability of the study on a national level, or have limitations based on population choice, such as older Medicare patients with fee-for-service coverage, so results may not be generalizable to younger patients or those with other types of insurance.^9,10^

Thus, a critical knowledge gap remains regarding whether breast cancer–specific survival disparities persist after accounting for individual, tumor, and treatment characteristics in a national, population-based cohort. This study fills this gap by evaluating the associations of neighborhood disadvantage, measured by a robust index of neighborhood disadvantage (Yost index), with breast cancer–specific and overall survival after accounting for individual, tumor, and treatment factors historically associated with worse survival (eg, Black race, insurance status, later stage at diagnosis, and lack of treatment) in a national cohort. We hypothesize that persistent survival disparities will remain, suggesting unaccounted-for mechanisms through which neighborhood disadvantage is associated with shorter breast cancer–specific survival.

## Methods

### Population and Data

We used prospective, population-based, deidentified cohort data from the 2016 to 2018 Surveillance, Epidemiology, and End Results (SEER) 17 database of the National Cancer Institute. The SEER program is a collection of population-based central cancer registries capturing facts from 21 geographic areas representing 36.7% of the US population.^11,12^ With permission from the SEER program, a specialized SEER dataset, which included additional information about area-based (Census tract–level) measures reflecting socioeconomic status and urban or rural residence, was used for these analyses. We included patients diagnosed with breast cancer stages I through IV between 2016 and 2018 (eFigure in Supplement 1). The Washington University School of Medicine institutional review board deemed this study not human participants research and waived the need for informed consent because data were deidentified. This study adhered to the Strengthening the Reporting of Observational Studies in Epidemiology (STROBE) reporting guidelines.^13^

### Primary Exposure/Outcome

Neighborhood disadvantage as measured through the Yost index was our primary exposure. The Yost index is the National Cancer Institute’s Census tract–level neighborhood socioeconomic index. The Yost index is a composite variable that includes Census tract–level variables: median household income, median house value, median rent, percentage below 150% of the poverty line, education index, percentage working class, and percentage unemployed.^14^ The Yost index was presented in quintiles representing the lowest to highest neighborhood socioeconomic indices. The Yost US-based quintile facilitates SES comparison across the US.^14^ The primary outcome was breast cancer–specific mortality. The secondary outcome was all-cause mortality.

### Covariates

Covariates were chosen based on current literature and subject matter knowledge to evaluate the association between neighborhood disadvantage and survival.^3,8,9,15,16^ For example, we controlled for race and ethnicity as a proxy for structural racism and used stage at presentation, insurance, rurality, and receipt of treatment to control for domains of access to care barriers.^3,9,15^ The final set of covariates included age at diagnosis, race and ethnicity, insurance status, rurality, stage at presentation, tumor subtype, and treatment receipt (surgery, radiation, and chemotherapy).

Age at diagnosis was categorized by decades. Self-identified race and ethnicity were categorized into American Indian/Alaskan Native, Asian, Hispanic, non-Hispanic Black, non-Hispanic White, and Pacific Islander. Race and ethnicity were provided by the SEER database, which collects these variables from Census survey data. Insurance status also served as a proxy for individual socioeconomic status.^9^ Rurality was evaluated through the rural-urban commuting area codes classified by US Census tracts using population density, urbanization, and daily commuting measures and was categorized into mostly urban, mostly rural, and all rural groups. The clinical stage at diagnosis was treated as a categorical variable and categorized as local, regional, distant, or unknown. Data were stratified by tumor subtype categorized based on breast cancer receptors (Luminal A, Luminal B, ERBB2 enriched, and triple-negative). To account for treatment, we used data on the completion of surgery, chemotherapy, and radiation evaluated for each patient.

### Statistical Analysis

We calculated descriptive statistics for all covariates as frequencies for the categorical variables and reported as mean (SD) for continuous variables. Pearson χ^2^ tests and analysis of variance were used, as appropriate, for intergroup bivariate analysis. Overall survival was evaluated using the Kaplan-Meier curve and log-rank test for *P* values. We calculated adjusted hazard ratios with a Cox proportional hazards regression for overall survival. To account for competing risks, the Fine-Gray subdistribution hazard model was calculated to analyze breast cancer–specific survival.^17^ Additionally, we used ArcGIS to geospatially map Yost index quintiles across the nation. All statistical tests were 2-sided, and statistical significance was assessed at an α less than .05. All data were analyzed in R, version 4.2.3 (R Project for Statistical Computing) with use of the survival package in R for survival analysis. Data were analyzed from September 2022 to December 2023.

## Results

### Patient Demographics

**Figure 1.**
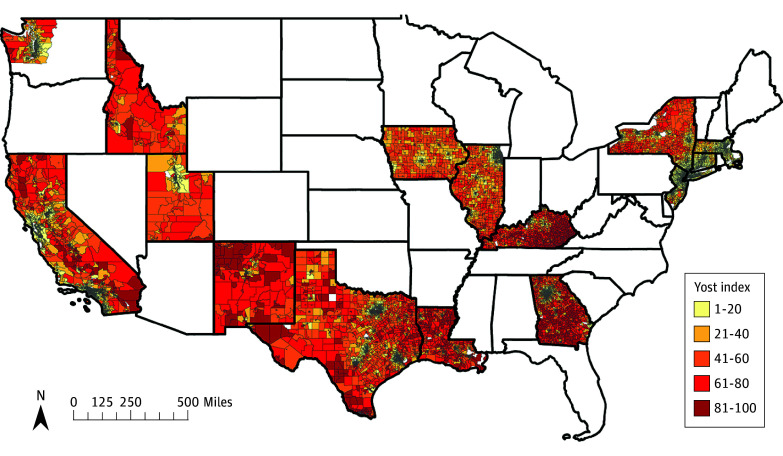
National Geospatial Distribution of Yost Index Quintiles Across the US

Overall, we identified 350 824 patients with breast cancer. A total of 41 519 (11.8%) were Hispanic, 39 631 (11.3%) were non-Hispanic Black, and 234 698 (66.9%) were non-Hispanic White. Patients were divided by Yost index quintiles, with 25.0% (87 635 patients) in the most advantaged neighborhoods (group 5) and 14.9% (52 439 patients) of the population living in the most disadvantaged neighborhoods (group 1). The distribution of Yost index quintiles across the nation can be seen in Figure 1. The most advantaged neighborhood had the highest proportion of non-Hispanic White patients (66 529 patients [76.2%]) while the most disadvantaged neighborhoods had the highest proportion of non-Hispanic Black (16 141 patients [30.9%]) and Hispanic patients (10 168 patients [19.5%]). Those living in the most disadvantaged neighborhoods (group 1) had the highest proportions of Medicaid insurance users, uninsured patients, and divorced, separated, or single status (Table 1).

**Table 1.** Patient Characteristics by Yost Index Quintiles

Characteristic	Group 1 (n = 52 439)	Group 2 (n = 62 402)	Group 3 (n = 70 414)	Group 4 (n = 77 934)	Group 5 (n = 87 635)	*P* value
Age, y						
<30	431 (0.8)	429 (0.7)	440 (0.6)	448 (0.6)	413 (0.5)	<.001
30-34	926 (1.8)	1052 (1.7)	1098 (1.6)	1200 (1.5)	1102 (1.3)	
35-39	1726 (3.3)	1911 (3.1)	2253 (3.2)	2479 (3.2)	2738 (3.1)	
40-44	3251 (6.2)	3766 (6.0)	4313 (6.1)	5148 (6.6)	6201 (7.1)	
45-49	4925 (9.4)	5699 (9.1)	6562 (9.3)	7756 (10.0)	10 124 (11.6)	
50-54	6162 (11.8)	7075 (11.3)	8102 (11.5)	9309 (11.9)	11 508 (13.1)	
55-59	6789 (12.9)	7964 (12.8)	8818 (12.5)	9868 (12.7)	10 819 (12.3)	
60-64	7267 (13.9)	8549 (13.7)	9699 (13.8)	10 821 (13.9)	11 584 (13.2)	
65-69	6683 (12.7)	8255 (13.2)	9481 (13.5)	10 310 (13.2)	11 180 (12.8)	
70-74	5224 (10.0)	6407 (10.3)	7119 (10.1)	7695 (9.9)	8283 (9.5)	
75-79	3963 (7.6)	4782 (7.7)	5180 (7.4)	5366 (6.9)	5624 (6.4)	
80-84	2665 (5.1)	3373 (5.4)	3821 (5.4)	3769 (4.8)	4059 (4.6)	
≥85	2427 (4.6)	3140 (5.0)	3528 (5.0)	3765 (4.8)	4000 (4.6)	
Year of diagnosis						
2010	6869 (13.1)	8302 (13.3)	9489 (13.5)	10 562 (13.6)	11 935 (13.6)	.02
2011	7149 (13.6)	8637 (13.8)	9713 (13.8)	11 010 (14.1)	12 248 (14.0)	
2012	7351 (14.0)	8821 (14.1)	9900 (14.1)	11 124 (14.3)	12 349 (14.1)	
2013	7550 (14.4)	8888 (14.2)	10 173 (14.4)	11 315 (14.5)	12 538 (14.3)	
2014	7666 (14.6)	8971 (14.4)	10 246 (14.6)	11 183 (14.3)	12 663 (14.4)	
2015	7889 (15.0)	9417 (15.1)	10 474 (14.9)	11 435 (14.7)	13 069 (14.9)	
2016	7965 (15.2)	9366 (15.0)	10 419 (14.8)	11 305 (14.5)	12 833 (14.6)	
Race and ethnicity						
American Indian or Alaska Native	236 (0.5)	315 (0.5)	317 (0.5)	265 (0.3)	193 (0.2)	<.001
East Asian	542 (1.0)	949 (1.5)	1670 (2.4)	2734 (3.5)	4896 (5.6)	
Hispanic	10 168 (19.5)	9440 (15.2)	8750 (12.5)	7320 (9.5)	5841 (6.7)	
Indian or Pakistani Asian	156 (0.3)	257 (0.4)	420 (0.6)	729 (0.9)	1625 (1.9)	
Non-Hispanic Black	16 141 (30.9)	8879 (14.3)	6872 (9.8)	4871 (6.3)	2868 (3.3)	
Non-Hispanic White	23 607 (45.2)	39 716 (64.1)	48 367 (69.1)	56 749 (73.4)	66 259 (76.2)	
Other Asian^a^	292 (0.6)	544 (0.9)	828 (1.2)	1186 (1.5)	1912 (2.2)	
Pacific Islander	172 (0.3)	357 (0.6)	568 (0.8)	671 (0.9)	549 (0.6)	
Southeast Asian	889 (1.7)	1541 (2.5)	2169 (3.1)	2823 (3.6)	2762 (3.2)	
Missing	236	404	453	586	730	
Insurance status						
Any Medicaid	13 381 (26.4)	10 114 (16.7)	8011 (11.8)	6113 (8.1)	4097 (4.8)	<.001
Insured	35 630 (70.3)	48 813 (80.8)	59 017 (86.6)	68 605 (90.6)	80 643 (94.2)	
Uninsured	1686 (3.3)	1472 (2.4)	1143 (1.7)	999 (1.3)	823 (1.0)	
Missing	1742	2003	2243	2217	2072	
Marital status						
Divorced or separated	7785 (15.9)	8211 (14.0)	8549 (12.9)	8643 (11.8)	7725 (9.2)	<.001
Married or domestic partner	21 081 (42.9)	30 560 (52.2)	37 607 (56.8)	44 545 (60.6)	56 686 (67.8)	
Single (never married)	11 759 (24.0)	10 358 (17.7)	10 157 (15.3)	10 588 (14.4)	9801 (11.7)	
Widowed	8469 (17.3)	9471 (16.2)	9882 (14.9)	9680 (13.2)	9345 (11.2)	
Missing	3345	3802	4219	4478	4078	
Rural urban index						
All urban	34 729 (66.2)	38 755 (62.1)	46 958 (66.7)	56 228 (72.1)	66 303 (75.7)	<.001
All rural	4800 (9.2)	6131 (9.8)	4097 (5.8)	1853 (2.4)	427 (0.5)	
Mostly rural	3332 (6.4)	5575 (8.9)	5474 (7.8)	4003 (5.1)	2604 (3.0)	
Mostly urban	9578 (18.3)	11 941 (19.1)	13 885 (19.7)	15 850 (20.3)	18 301 (20.9)	
Tumor subtype						
HR− and ERBB2− (triple negative)	7050 (13.4)	7026 (11.3)	7411 (10.5)	7468 (9.6)	7383 (8.4)	<.001
HR− and ERBB2+ (ERBB2 enriched)	2653 (5.1)	2887 (4.6)	2984 (4.2)	3352 (4.3)	3467 (4.0)	
HR+ and ERBB2− (luminal A)	31 835 (60.7)	40 162 (64.4)	47 059 (66.8)	53 294 (68.4)	61 842 (70.6)	
HR+ and ERBB2+ (luminal B)	5467 (10.4)	6626 (10.6)	7175 (10.2)	7771 (10.0)	8664 (9.9)	
Unknown	5434 (10.4)	5701 (9.1)	5785 (8.2)	6049 (7.8)	6279 (7.2)	
Stage						
Distant	5119 (7.2)	5365 (6.1)	5611 (5.5)	5739 (5.0)	5584 (4.2)	<.001
Localized	42 563 (59.7)	55 729 (63.1)	66 047 (65.0)	76 572 (66.5)	91 214 (69.0)	
Regional	22 877 (32.1)	26 437 (29.9)	29 256 (28.8)	31 949 (27.8)	34 768 (26.3)	
Missing	722 (1.0)	743 (0.8)	721 (0.7)	812 (0.7)	696 (0.5)	
Surgery						
No	6613 (12.8)	6643 (10.7)	6736 (9.6)	7151 (9.2)	7162 (8.2)	<.001
Yes	45 228 (87.2)	55 215 (89.3)	63 120 (90.4)	70 207 (90.8)	79 983 (91.8)	
Radiotherapy						
No or unknown	26 672 (52.7)	30 218 (50.1)	32 591 (47.9)	35 399 (47.0)	39 637 (46.7)	<.001
Yes	23 892 (47.3)	30 042 (49.9)	35 457 (52.1)	39 886 (53.0)	45 213 (53.3)	
Chemotherapy						
No or unknown	29 505 (56.3)	36 414 (58.4)	42 062 (59.7)	47 444 (60.9)	54 394 (62.1)	<.001
Yes	22 934 (43.7)	25 988 (41.6)	28 352 (40.3)	30 490 (39.1)	33 241 (37.9)	

Abbreviations: +, positive; −, negative; HR, hormone receptor.

^a^ Other Asian is a race category provided by SEER including Asian, not otherwise specified and Oriental, not otherwise specified.

### Tumor and Treatment Characteristics

Patients in the most disadvantaged neighborhoods had the highest proportion of triple-negative breast cancer (7050 patients [13.4%]) and regional (22 877 patients [32.1%]) and distant (5119 patients [7.2%]) disease stage compared with other Yost index groups (Table 1). Patients in this group also had the lowest proportion of patients to complete surgery (45 228 patients [87.2%]) and radiation (23 892 patients [47.3%]) and the highest proportion of patients that received chemotherapy (22 934 patients [43.7%]) compared with all other Yost index groups. The most advantaged neighborhoods had higher proportions of localized stage (91 214 patients [69.0%]) and luminal A (61 842 patients [70.6%]) tumor subtype. These patients had a higher proportion of surgery (79 983 patients [91.8%]) and radiation (45 213 patients [53.3%]) and the lowest proportion of chemotherapy treatment (33 241 patients [37.9%]) compared with less advantaged groups.

### Survival Analysis

**Figure 2.**
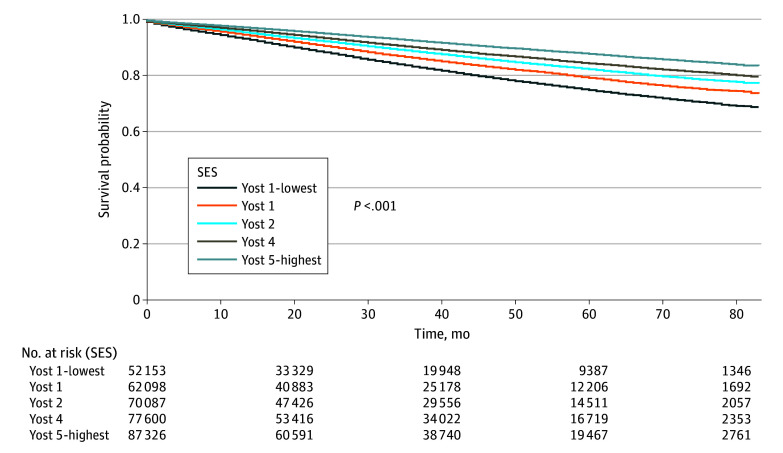
Kaplan-Meier Curves for Overall Survival by Yost Index Quintiles

Overall, there were 323 510 patients included in the survival analysis. There were 27 145 breast cancer–specific deaths and 17 345 non–breast cancer deaths. On Kaplan-Meier analysis, overall survival was significantly different among Yost index groups (*P* < .001) (Figure 2). For the multivariate overall survival analysis, after adjusting for age, race and ethnicity, rurality, stage, subtype, insurance, and receipt of treatment (surgery, chemotherapy, and radiation), patients in the most disadvantaged neighborhoods (group 1) had the highest risk of mortality (HR, 1.53; 95% CI, 1.48-1.59; *P* < .001) compared with the most advantaged neighborhoods (group 5) (Table 2). Non-Hispanic Black patients had the highest risk of mortality compared with non-Hispanic White patients (HR, 1.16; 95% CI, 1.13-1.20; *P* < .001).

**Table 2.** Hazard Ratios for Overall Survival and Breast Cancer–Specific Survival

Characteristic	Overall survival		Breast cancer–specific survival	
	Hazard ratio (95% CI)	*P* value	Hazard ratio (95% CI)	*P* value
Yost index				
Group 1	1.53 (1.48-1.59)	<.001	1.43 (1.36-1.50)	<.001
Group 2	1.45 (1.40-1.50)	<.001	1.37 (1.30-1.43)	<.001
Group 3	1.28 (1.24-1.32)	<.001	1.24 (1.18-1.30)	<.001
Group 4	1.19 (1.15-1.23)	<.001	1.17 (1.11-1.22)	<.001
Group 5	1 [Reference]	NA	1 [Reference]	NA
Race and ethnicity			0.00 (0.00-0.00)	
American Indian or Alaska Native	1.06 (0.90-1.25)	.47	0.92 (0.73-1.17)	.51
Asian	0.73 (0.70-0.77)	<.001	0.82 (0.77-0.87)	<.001
Hispanic	0.86 (0.83-0.89)	<.001	0.93 (0.88-0.97)	.002
Non-Hispanic Black	1.16 (1.13-1.20)	<.001	1.16 (1.11-1.22)	<.001
Non-Hispanic White	1 [Reference]	NA	1 [Reference]	NA
Pacific Islander	1.10 (0.96-1.25)	.16	1.10 (0.92-1.31)	.30
Age, y				
<30	0.74 (0.64-0.86)	<.001	0.84 (0.71-1.00)	.05
30-34	0.85 (0.78-0.94)	.001	1.01 (0.91-1.12)	.90
35-39	0.80 (0.74-0.86)	<.001	0.98 (0.90-1.06)	.57
40-44	0.73 (0.69-0.78)	<.001	0.92 (0.86-0.99)	.02
45-49	0.70 (0.66-0.74)	<.001	0.85 (0.80-0.91)	<.001
50-54	0.81 (0.77-0.85)	<.001	0.96 (0.90-1.01)	.12
55-59	0.93 (0.88-0.97)	.001	1.01 (0.96-1.07)	.63
60-64	1 [Reference]	NA	1 [Reference]	NA
65-69	1.22 (1.17-1.28)	<.001	1.06 (1.00-1.13)	.04
70-74	1.56 (1.49-1.64)	<.001	1.22 (1.14-1.29)	<.001
75-79	2.16 (2.06-2.26)	<.001	1.47 (1.38-1.57)	<.001
80-84	3.23 (3.08-3.38)	<.001	1.79 (1.67-1.92)	<.001
≥85	5.13 (4.91-5.36)	<.001	2.46 (2.29-2.65)	<.001
Rural urban index				
All rural	1.02 (0.98-1.07)	.36	0.99 (0.92-1.06)	.74
All urban	1 [Reference]	NA	1 [Reference]	NA
Mostly rural	1.03 (0.99-1.08)	.16	1.03 (0.97-1.10)	.35
Mostly urban	1.01 (0.98-1.04)	.42	0.97 (0.93-1.00)	.08
Insurance			0.00 (0.00-0.00)	
Any Medicaid	1.53 (1.48-1.57)	<.001	1.34 (1.29-1.39)	<.001
Insured	1 [Reference]	NA	1 [Reference]	NA
Uninsured	1.67 (1.57-1.78)	<.001	1.47 (1.36-1.60)	<.001
Tumor subtype			0.00 (0.00-0.00)	
HR- and ERBB2− (triple negative)	2.76 (2.68-2.84)	<.001	3.20 (3.06-3.35)	<.001
HR- and ERBB2+ (ERBB2 enriched)	1.47 (1.41-1.55)	<.001	1.52 (1.43-1.62)	<.001
HR+ and ERBB2− (luminal A)	1 [Reference]	NA	1 [Reference]	NA
HR+ and ERBB2+ (luminal B)	1.03 (0.99-1.07)	.20	1.02 (0.97-1.07)	.42
Unknown	1.51 (1.46-1.56)	<.001	1.45 (1.36-1.54)	<.001
Stage				
Distant	8.23 (7.95-8.51)	<.001	16.84 (15.88-17.85)	<.001
Localized	1 [Reference]	NA	1 [Reference]	NA
Regional	2.47 (2.41-2.54)	<.001	4.29 (4.12-4.47)	<.001
Surgery				
Yes	0.36 (0.35-0.37)	<.001	0.36 (0.35-0.38)	<.001
No	1 [Reference]	NA	1 [Reference]	NA
Radiation				
Yes	0.70 (0.68-0.72)	<.001	0.81 (0.78-0.83)	<.001
No	1 [Reference]	NA	1 [Reference]	NA
Chemotherapy				
Yes	0.86 (0.84-0.88)	<.001	1.04 (1.00-1.08)	.07
No	1 [Reference]	NA	1 [Reference]	NA

Abbreviations: +, positive; −, negative; HR, hormone receptor; NA, not applicable.

Our findings held when considering competing risks for breast cancer–specific survival and controlling for the same covariates. The most disadvantaged neighborhoods (group 1) had the highest risk of mortality (HR, 1.43; 95% CI, 1.36-1.50; *P* < .001) compared with the most advantaged neighborhoods (Table 2). Similarly, non-Hispanic Black patients continued to have the highest risk of mortality among racial and ethnic groups compared with non-Hispanic White patients (HR, 1.16; 95% CI, 1.11-1.22; *P* < .001). Restricted mean survival times were calculated by group with increasingly shorter survival as neighborhood disadvantage increased by Yost groups and can be viewed in Table 3. The time horizon was 83 months.

**Table 3.** Restricted Mean Survival Time (RMST) by Yost Index Quintiles

Yost index quintile	RMST (LCL to UCL)	Change in RMST (LCL to UCL)	*P* value
Group 1	68.2 (67.9 to 68.5)	−7.7 (−8.0 to −7.4)	<.001
Group 2	70.8 (70.6 to 71.0)	−5.1 (−5.4 to −4.8)	<.001
Group 3	72.6 (72.4 to 72.8)	−3.3 (−3.5 to −3.0)	<.001
Group 4	74.0 (73.8 to 74.1)	−1.9 (−2.2 to −1.7)	<.001
Group 5	75.9 (75.7 to 76.1)	1 [Reference]	NA

Abbreviations: LCL, lower confidence limit; NA, not applicable; UCL, upper confidence limit.

## Discussion

This cohort study discovered that patients in a population-based national cohort living in the most disadvantaged neighborhoods had shorter breast cancer–specific and overall survival, even after adjusting for individual, tumor, and treatment characteristics that are typically used to explain survival differences. This persistent effect of neighborhood disadvantage on survival suggests potential unaccounted-for biological and nonbiological mechanisms by which neighborhood disadvantage influences shorter breast cancer–specific survival.^9,15,18,19,20,21^

Our study expands on the current literature in important ways. Other large national database studies assessing breast cancer–specific mortality have found similar associations with measures of neighborhood disadvantage; however, these studies were limited by either only assessing older Medicare patients^9^ or by not accounting for treatment, which limits our understanding of whether survival disparities persist even after controlling for treatment.^15^ A recent study by Abdel-Rahman et al^15^ in 2019 found that neighborhood disadvantage was associated with shortened breast cancer–specific survival in a national cohort; however, this study does not account for critical social determinants of health such as insurance and rurality, which are known to affect breast cancer outcomes.^3,22,23^ Additionally, prior studies did not include important minority racial and ethnic groups or oversimplified breast cancer receptor status. By using a specialized SEER dataset, we overcome prior limitations and capture a population-based breast cancer cohort which has not been previously analyzed through the lens of neighborhood-level disadvantage independent of individual-level risk, sociodemographic, access to care, tumor, and treatment factors. Moreover, geospatial mapping of Yost index quintiles exemplifies how we can use geospatial tools to identify areas for targeted interventions and cancer control programs.^24^

Our findings of persistent breast cancer–specific survival disparities by neighborhood disadvantage after adjusting for individual, tumor, and treatment characteristics suggests unaccounted-for mechanisms by which neighborhood influences survival.^20,25,26,27,28,29,30,31,32,33,34,35,36,37,38,39,40,41^ In addition to potential unmeasured social barriers, these findings raise the question of whether neighborhood disadvantage may be leading to more aggressive tumor biology and ultimately shorter breast cancer–specific survival. Social genomics studies have begun to evaluate how social adversity, such as neighborhood disadvantage, impacts tumor biology in cancer.^25,26,27,28,29^ In fact, neighborhood disadvantage has been associated with psychological and biological stress measures in humans including increased cortisol levels, allostatic load, and measures of biological aging.^25,30,31,32^ Psychological stressors such as crime and violence place demands on the sympathetic nervous system, causing upregulation of stress-related neuroendocrine signaling pathways. Social genomics studies have identified that these pathways lead to a social adversity–associated blood leukocyte gene expression termed the conserved transcriptional response to adversity (CTRA).^20,33,34,35,36,37^ CTRA gene expression has been studied in murine and human tissues in breast and other forms of cancer, and is associated with upregulation of proinflammatory gene expression, which fosters a prometastatic environment indicative of aggressive tumor biology.^38,39,40^ This social signal transduction pathway may explain how neighborhood disadvantage influences breast cancer survival by causing stress that leads to aggressive tumor biology. However, the relationship between neighborhood disadvantage and CTRA gene and tumor expression has yet to be established in humans and requires further study. The study of the biological effects of social determinants of health on tumor biology may provide targetable interventions to reduce disparities, along with policy change to dismantle complex causes of societal inequities.^41^

In addition to neighborhood disadvantage, we found that non-Hispanic Black patients had the highest mortality risk compared with non-Hispanic White patients and Hispanic patients. This potential residual disparity might be associated with social determinants of health that were not controlled for as well as germline genetics such as genetic ancestry. This is in line with previous studies that have found that West African ancestry is associated with more aggressive tumor subtypes and worse survival.^5,42,43,44,45^ Similar results have been discovered for relative risk of triple negative breast cancer (TNBC) compared with estrogen receptor positive, human epidermal growth factor receptor 2 negative (ERBB2−) disease. A recent study by Goel et al^8^ identified that both low neighborhood socioeconomic status and increasing West African genetic ancestry (both global and local) are associated with higher odds of TNBC on univariable analysis; however, neighborhood socioeconomic status remained an independent predictor of TNBC on multivariable analysis, suggesting that socioeconomic status may impact higher odds of TNBC.^16,42,44,46^ Linnenbringer et al,^6^ using population-based California Cancer Registry data, found that among Black women, both higher neighborhood income and higher percentages of Black neighborhood residents were associated with lower odds of TNBC relative to hormone receptor positive /ERBB2− disease. Combined, these studies highlight that the associations between neighborhood composition, neighborhood socioeconomic status, and odds of TNBC differ by self-identified race and ethnicity, genetic ancestry, and age. Controlling for race and ethnicity strengthens the argument that neighborhoods themselves, larger structures that promote inequities for minority groups, are associated with disparities across all races and ethnicities. In other words, by controlling for individual-level race or ethnicity, we can shift the focus of research toward targetable, structural changes instead of racialized-biological differences.^47,48,49^

In addition to hypothesized biologic mechanisms, nonbiologic pathways not accounted for in our analysis may also be contributing to persistent disparities in breast cancer–specific survival. Attitudes toward and trust of health care practitioners,^50^ financial burden,^51^ and the immeasurable effects of structural racism^47^ that pervade our systems of health care, education, employment, insurance, and the justice system, all contribute to inequities in survival and must be appropriately accounted for in future national studies.^8,16^

### Limitations

This study has limitations. Beyond the limitations of retrospective observational studies, SEER lacks information on comorbidities as well as detailed information about treatment rationale, doses, and completion of chemotherapy and radiotherapy. Another limitation is that although we used individual insurance coverage as a proxy for access to care, this does not completely represent all access to care measures.

## Conclusions

In this population-based national cohort study, we found that neighborhood disadvantage is independently associated with shorter survival in patients with breast cancer even after controlling for individual-level factors, tumor characteristics, and treatment. To address these residual disparities associated with neighborhood disadvantage, research must focus on which components of the built environment influence outcomes. To address these residual disparities associated with neighborhood disadvantage, future research must take a translational epidemiologic approach to focus on biological and nonbiological factors through which the built environment, above the beyond individual-level factors, may influence outcomes. This approach to research has the potential to advance precision medicine in oncology by bringing neighborhood disadvantage into consideration when risk-stratifying vulnerable populations and to develop cancer control interventions to overcome breast cancer disparities.

## Notes

Supplement 1. **eFigure.** Study Flow Diagram

Supplement 2.
Data Sharing Statement

## References

[1] Krieger N, Kim R, Feldman J, Waterman PD. Using the index of concentration at the extremes at multiple geographical levels to monitor health inequities in an era of growing spatial social polarization: Massachusetts, USA (2010-14). Int J Epidemiol. 2018;47(3):788-819. doi:10.1093/ije/dyy004 29522187

[2] Gill TM, Zang EX, Murphy TE, . Association between neighborhood disadvantage and functional well-being in community-living older persons. JAMA Intern Med. 2021;181(10):1297-1304. doi:10.1001/jamainternmed.2021.4260 34424276 PMC8383163

[3] Coughlin SS. Social determinants of breast cancer risk, stage, and survival. Breast Cancer Res Treat. 2019;177(3):537-548. doi:10.1007/s10549-019-05340-7 31270761

[4] Dietze EC, Sistrunk C, Miranda-Carboni G, O’Regan R, Seewaldt VL. Triple-negative breast cancer in African-American women: disparities versus biology. Nat Rev Cancer. 2015;15(4):248-254. doi:10.1038/nrc3896 25673085 PMC5470637

[5] Newman LA, Kaljee LM. Health disparities and triple-negative breast cancer in African American Women: a review. JAMA Surg. 2017;152(5):485-493. doi:10.1001/jamasurg.2017.0005 28355428

[6] Linnenbringer E, Geronimus AT, Davis KL, Bound J, Ellis L, Gomez SL. Associations between breast cancer subtype and neighborhood socioeconomic and racial composition among Black and White women. Breast Cancer Res Treat. 2020;180(2):437-447. doi:10.1007/s10549-020-05545-1 32002766 PMC7066090

[7] Goel N, Hernandez A, Thompson C, . Neighborhood disadvantage and breast cancer-specific survival. JAMA Netw Open. 2023;6(4):e238908. doi:10.1001/jamanetworkopen.2023.8908 37083666 PMC10122178

[8] Goel N, Westrick AC, Bailey ZD, . Structural racism and breast cancer-specific survival: impact of economic and racial residential segregation. Ann Surg. 2022;275(4):776-783. doi:10.1097/SLA.0000000000005375 35081560 PMC9102835

[9] Cheng E, Soulos PR, Irwin ML, . Neighborhood and individual socioeconomic disadvantage and survival among patients with nonmetastatic common cancers. JAMA Netw Open. 2021;4(12):e2139593. doi:10.1001/jamanetworkopen.2021.39593 34919133 PMC8683967

[10] Luningham JM, Seth G, Saini G, . Association of race and area deprivation with breast cancer survival among Black and White women in the state of Georgia. JAMA Netw Open. 2022;5(10):e2238183. doi:10.1001/jamanetworkopen.2022.38183 36306134 PMC9617173

[11] National Cancer Institute. SEER*Stat Databases. Accessed December 15, 2022. https://seer.cancer.gov/data-software/documentation/seerstat/

[12] Siegel RL, Miller KD, Jemal A. Cancer statistics, 2020. CA Cancer J Clin. 2020;70(1):7-30. doi:10.3322/caac.21590 31912902

[13] Vandenbroucke JP, von Elm E, Altman DG, ; STROBE Initiative. Strengthening the Reporting of Observational Studies in Epidemiology (STROBE): explanation and elaboration. PLoS Med. 2007;4(10):e297. doi:10.1371/journal.pmed.0040297 17941715 PMC2020496

[14] Yost K, Perkins C, Cohen R, Morris C, Wright W. Socioeconomic status and breast cancer incidence in California for different race/ethnic groups. Cancer Causes Control. 2001;12(8):703-711. doi:10.1023/A:1011240019516 11562110

[15] Abdel-Rahman O. Impact of NCI Socioeconomic Index on the outcomes of nonmetastatic breast cancer patients: analysis of SEER census tract-level socioeconomic database. Clin Breast Cancer. 2019;19(6):e717-e722. doi:10.1016/j.clbc.2019.06.013 31519450

[16] Goel N, Yadegarynia S, Lubarsky M, . Racial and ethnic disparities in breast cancer survival: emergence of a clinically distinct Hispanic Black population. Ann Surg. 2021;274(3):e269-e275. doi:10.1097/SLA.0000000000005004 34132699 PMC8384141

[17] Austin PC, Fine JP. Practical recommendations for reporting Fine-Gray model analyses for competing risk data. Stat Med. 2017;36(27):4391-4400. doi:10.1002/sim.7501 28913837 PMC5698744

[18] Bhattacharyya O, Li Y, Fisher JL, . Low neighborhood socioeconomic status is associated with higher mortality and increased surgery utilization among metastatic breast cancer patients. Breast. 2021;59:314-320. doi:10.1016/j.breast.2021.08.003 34388697 PMC8361177

[19] Shariff-Marco S, Yang J, John EM, . Impact of neighborhood and individual socioeconomic status on survival after breast cancer varies by race/ethnicity: the Neighborhood and Breast Cancer Study. Cancer Epidemiol Biomarkers Prev. 2014;23(5):793-811. doi:10.1158/1055-9965.EPI-13-0924 24618999 PMC4018239

[20] Saini G, Ogden A, McCullough LE, Torres M, Rida P, Aneja R. Disadvantaged neighborhoods and racial disparity in breast cancer outcomes: the biological link. Cancer Causes Control. 2019;30(7):677-686. doi:10.1007/s10552-019-01180-4 31111277 PMC7043809

[21] Smith BP, Madak-Erdogan Z. Urban neighborhood and residential factors associated with breast cancer in African American women: a systematic review. Horm Cancer. 2018;9(2):71-81. doi:10.1007/s12672-018-0325-x 29417390 PMC10355946

[22] Wang Z, Dong Y, Lu Z, Chen Z. Socioeconomic status variables contribute to the disparities in female triple negative breast cancer outcome in the United States, 2011-2015: a population study based on NCI Surveillance, Epidemiology and End Results (SEER) database. J Clin Oncol. 2021;39(15)(suppl):6521-6521. doi:10.1200/JCO.2021.39.15_suppl.6521

[23] Celaya MO, Rees JR, Gibson JJ, Riddle BL, Greenberg ER. Travel distance and season of diagnosis affect treatment choices for women with early-stage breast cancer in a predominantly rural population (United States). Cancer Causes Control. 2006;17(6):851-856. doi:10.1007/s10552-006-0025-7 16783613

[24] Mobley LR, Kuo TM, Scott L, Rutherford Y, Bose S. Modeling geospatial patterns of late-stage diagnosis of breast cancer in the US. Int J Environ Res Public Health. 2017;14(5):484. doi:10.3390/ijerph14050484 28475134 PMC5451935

[25] Hill TD, Ross CE, Angel RJ. Neighborhood disorder, psychophysiological distress, and health. J Health Soc Behav. 2005;46(2):170-186. doi:10.1177/002214650504600204 16028456

[26] Ross CE, Mirowsky J. Neighborhood disorder, subjective alienation, and distress. J Health Soc Behav. 2009;50(1):49-64. doi:10.1177/002214650905000104 19413134

[27] Rosenzweig MQ, Althouse AD, Sabik L, . The association between area deprivation index and patient-reported outcomes in patients with advanced cancer. Health Equity. 2021;5(1):8-16. doi:10.1089/heq.2020.0037 33564735 PMC7868579

[28] Antoni MH, Lutgendorf SK, Cole SW, . The influence of bio-behavioural factors on tumour biology: pathways and mechanisms. Nat Rev Cancer. 2006;6(3):240-248. doi:10.1038/nrc1820 16498446 PMC3146042

[29] Chang A, Sloan EK, Antoni MH, Knight JM, Telles R, Lutgendorf SK. Biobehavioral pathways and cancer progression: insights for improving well-being and cancer outcomes. Integr Cancer Ther. Published online May 17, 2022. doi:10.1177/15347354221096081 35579197 PMC9118395

[30] Serrano-Gomez SJ, Sanabria-Salas MC, Fejerman L. Breast cancer health disparities in Hispanics/Latinas. Curr Breast Cancer Rep. 2020;12(3):175-184. doi:10.1007/s12609-020-00370-3 33584971 PMC7879708

[31] Costanzo ES, Sood AK, Lutgendorf SK. Biobehavioral influences on cancer progression. Immunol Allergy Clin North Am. 2011;31(1):109-132. doi:10.1016/j.iac.2010.09.001 21094927 PMC3011980

[32] Smith JA, Zhao W, Wang X, . Neighborhood characteristics influence DNA methylation of genes involved in stress response and inflammation: the multi-ethnic study of atherosclerosis. Epigenetics. 2017;12(8):662-673. doi:10.1080/15592294.2017.1341026 28678593 PMC5687339

[33] Lee MJ, Rittschof CC, Greenlee AJ, . Transcriptomic analyses of black women in neighborhoods with high levels of violence. Psychoneuroendocrinology. 2021;127:105174. doi:10.1016/j.psyneuen.2021.105174 33647572 PMC9191231

[34] Antoni MH, Dhabhar FS. The impact of psychosocial stress and stress management on immune responses in patients with cancer. Cancer. 2019;125(9):1417-1431. doi:10.1002/cncr.31943 30768779 PMC6467795

[35] McEwen BS. Sex, stress and the hippocampus: allostasis, allostatic load and the aging process. Neurobiol Aging. 2002;23(5):921-939. doi:10.1016/S0197-4580(02)00027-1 12392796

[36] Simons RL, Lei MK, Klopack E, Zhang Y, Gibbons FX, Beach SRH. Racial discrimination, inflammation, and chronic illness among African American Women at midlife: support for the weathering perspective. J Racial Ethn Health Disparities. 2021;8(2):339-349. doi:10.1007/s40615-020-00786-8 32488825 PMC8183614

[37] Cole SW. The conserved transcriptional response to adversity. Curr Opin Behav Sci. 2019;28:31-37. doi:10.1016/j.cobeha.2019.01.008 31592179 PMC6779418

[38] Cole SW, Nagaraja AS, Lutgendorf SK, Green PA, Sood AK. Sympathetic nervous system regulation of the tumour microenvironment. Nat Rev Cancer. 2015;15(9):563-572. doi:10.1038/nrc3978 26299593 PMC4828959

[39] Sloan EK, Priceman SJ, Cox BF, . The sympathetic nervous system induces a metastatic switch in primary breast cancer. Cancer Res. 2010;70(18):7042-7052. doi:10.1158/0008-5472.CAN-10-0522 20823155 PMC2940980

[40] Lutgendorf SK, Penedo F, Goodheart MJ, . Epithelial-mesenchymal transition polarization in ovarian carcinomas from patients with high social isolation. Cancer. 2020;126(19):4407-4413. doi:10.1002/cncr.33060 32691853 PMC7719066

[41] Carlos RC, Obeng-Gyasi S, Cole SW, . Linking structural racism and discrimination and breast cancer outcomes: a social genomics approach. J Clin Oncol. 2022;40(13):1407-1413. doi:10.1200/JCO.21.02004 35108027 PMC9851699

[42] Goel N, Yadegarynia S, Kwon D, . Translational epidemiology: an integrative approach to determine the interplay between genetic ancestry and neighborhood socioeconomic status on triple negative breast cancer. Ann Surg. 2022;276(3):430-440. doi:10.1097/SLA.0000000000005554 35758508 PMC11611250

[43] Iyer HS, Gomez SL, Cheng I, Rebbeck TR. Relative impact of genetic ancestry and neighborhood socioeconomic status on all-cause mortality in self-identified African Americans. PLoS One. 2022;17(8):e0273735. doi:10.1371/journal.pone.0273735 36037186 PMC9423617

[44] Jiagge E, Jibril AS, Chitale D, . Comparative analysis of breast cancer phenotypes in African American, White American, and West versus East African patients: correlation between African ancestry and triple-negative breast cancer. Ann Surg Oncol. 2016;23(12):3843-3849. doi:10.1245/s10434-016-5420-z 27469125

[45] Martini R, Delpe P, Chu TR, . African ancestry-associated gene expression profiles in triple-negative breast cancer underlie altered tumor biology and clinical outcome in women of African descent. Cancer Discov. 2022;12(11):2530-2551. doi:10.1158/2159-8290.CD-22-0138 36121736 PMC9627137

[46] Davis M, Martini R, Newman L, . Identification of distinct heterogenic subtypes and molecular signatures associated with African ancestry in triple negative breast cancer using quantified genetic ancestry models in admixed race populations. Cancers (Basel). 2020;12(5):1220. doi:10.3390/cancers12051220 32414099 PMC7281131

[47] Bailey ZD, Feldman JM, Bassett MT. How structural racism works—racist policies as a root cause of U.S. Racial health inequities. N Engl J Med. 2021;384(8):768-773. doi:10.1056/NEJMms2025396 33326717 PMC11393777

[48] Bailey ZD, Krieger N, Agénor M, Graves J, Linos N, Bassett MT. Structural racism and health inequities in the USA: evidence and interventions. Lancet. 2017;389(10077):1453-1463. doi:10.1016/S0140-6736(17)30569-X 28402827

[49] Hoffman KM, Trawalter S, Axt JR, Oliver MN. Racial bias in pain assessment and treatment recommendations, and false beliefs about biological differences between blacks and whites. Proc Natl Acad Sci U S A. 2016;113(16):4296-4301. doi:10.1073/pnas.1516047113 27044069 PMC4843483

[50] Ahern MM, Hendryx MS. Social capital and trust in providers. Soc Sci Med. 2003;57(7):1195-1203. doi:10.1016/S0277-9536(02)00494-X 12899904

[51] Beltrán Ponce SE, Thomas CR, Diaz DA. Social determinants of health, workforce diversity, and financial toxicity: a review of disparities in cancer care. Curr Probl Cancer. 2022;46(5):100893. doi:10.1016/j.currproblcancer.2022.100893 35985886

