# Supplementary material for: Neighborhood Disadvantage and Breast Cancer–Specific Survival in the US
Source: JAMA Netw Open. 2024 Apr 18;7(4):e247336. doi: 10.1001/jamanetworkopen.2024.7336 (PMC12634134; doi:10.1001/jamanetworkopen.2024.7336)
Supplement: Supplement 1. — eFigure. Study Flow Diagram [file jamanetwopen-e247336-s001.pdf]

# Supplemental Online Content

Goel N, Hernandez A, Mazul A. Neighborhood disadvantage and breast cancer-specific survival in the US. *JAMA Netw Open*. 2024;7(4):e247336. doi:10.1001/jamanetworkopen.2024.7336

**eFigure.** Study Flow Diagram

This supplemental material has been provided by the authors to give readers additional information about their work.

© 2024 Goel N et al. *JAMA Network Open.*

**Figure S1.** Study Flow Diagram.

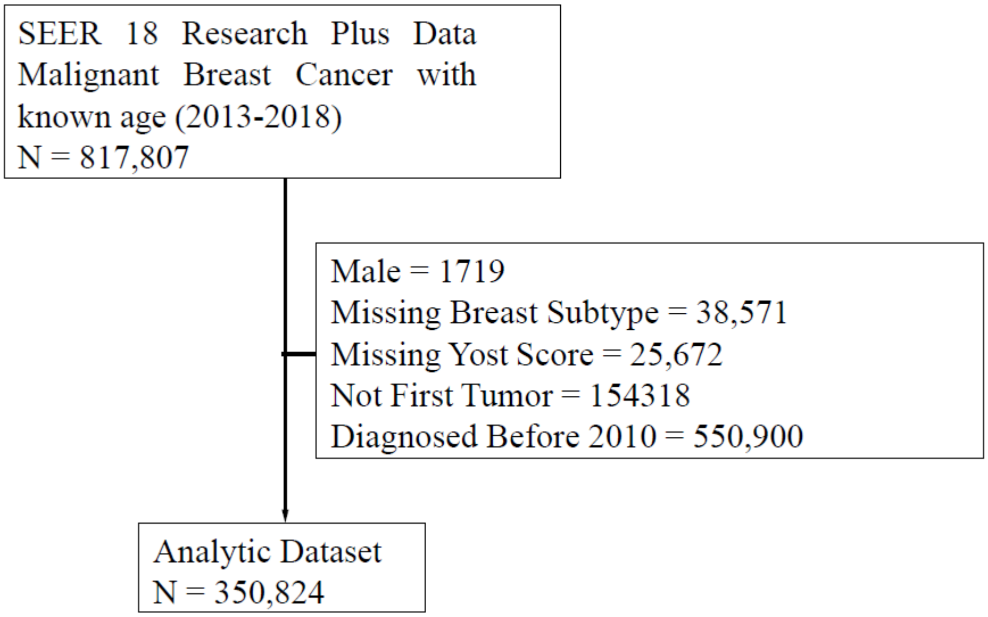


© 2024 Goel N et al. *JAMA Network Open.*
