# Supplementary material for: Neighborhood Disadvantage and Breast Cancer–Specific Survival in the US
Source: JAMA Netw Open. 2024 Apr 18;7(4):e247336. doi: 10.1001/jamanetworkopen.2024.7336 (PMC12634134; doi:10.1001/jamanetworkopen.2024.7336)
Supplement: Supplement 2. — Data Sharing Statement [file jamanetwopen-e247336-s002.pdf]

Data Sharing Statement

Goel. Neighborhood Disadvantage and Breast Cancer–Specific Survival in the US. *JAMA Netw Open*. Published April 18, 2024. doi:10.1001/jamanetworkopen.2024.7336

Data
Data available: No

Additional Information
Explanation for why data not available: Dataset is publicly available.
